# Hemorrhagic Coagulation Disorders and Ischemic Stroke: How to Reconcile Both?

**DOI:** 10.3390/neurolint15040093

**Published:** 2023-11-30

**Authors:** Pietro Crispino

**Affiliations:** Medicine Unit, Santa Maria Goretti Hospital, Via Scaravelli Snc, 04100 Latina, Italy; picrispino@libero.it

**Keywords:** stroke, emocoagulation, fibrinolysis

## Abstract

Coagulation and fibrinolytic system disorders are conditions in which the blood’s ability to clot is impaired, resulting in an increased risk of thrombosis or bleeding. Although these disorders are the expression of two opposing tendencies, they can often be associated with or be a consequence of each other, contributing to making the prognosis of acute cerebrovascular events more difficult. It is important to recognize those conditions that are characterized by dual alterations in the coagulation and fibrinolytic systems to reduce the prognostic impact of clinical conditions with difficult treatment and often unfortunate outcomes. Management of these individuals can be challenging, as clinicians must balance the need to prevent bleeding episodes with the potential risk of clot formation. Treatment decisions should be made on an individual basis, considering the specific bleeding disorder, its severity, and the patient’s general medical condition. This review aims to deal with all those forms in which coagulation and fibrinolysis represent two sides of the same media in the correct management of patients with acute neurological syndrome. Precision medicine, personalized treatment, advanced anticoagulant strategies, and innovations in bleeding control represent future directions in the management of these complex pathologies in which stroke can be the evolution of two different acute events or be the first manifestation of an occult or unknown underlying pathology.

## 1. Background

In the last decade, many steps forward have been made in the search for new anticoagulant drugs capable of acting on the pro-coagulative activity of some pathological conditions such as atrial fibrillation or congenital anomalies of the blood coagulation system (factor V deficiency Leiden, protein C, and protein S deficiency, hyperhomocysteinemia) [1,2]. New drugs have therefore been introduced, including direct anticoagulants, which are now key therapies in numerous pathological conditions and are added to antifibrinolytics, platelet inhibitors, and vitamin-K-dependent anticoagulants [1,2,3,4]. Their therapeutic benefits are indisputable; however, all evidence for these treatments must be balanced against the risk of bleeding complications, which are relatively frequent for some of these drugs. Warfarin, the oral anticoagulant that until a few years ago was the most commonly used, substantially increases the risk of major bleeding complications, including hemorrhagic stroke and gastrointestinal bleeding, by up to 3–8% per year [5,6]. Similarly, major bleeding is a serious and frequent complication of all inhibitors of platelet activity, including adenosine diphosphate 3–5 receptor antagonists such as clopidogrel and prasugrel [7,8]. We have therefore focused our attention more on the risk of ischemic stroke in hemorrhagic patients as it always represents a critical issue in the management of patients, especially the most fragile patients. This represents a condition in which it is always difficult to make a choice, with a consequent increase in mortality rates and health care costs in terms of worsening frailty if the patient were to survive. This review analyzes all the aspects linked to these antithetical conditions in order to better think about the appropriate therapies to apply to these patients as categories at greater risk of complications.

## 2. Stratification of Bleeding Risk in Patients Treated for Vascular Events Prevention

The occurrence of bleeding, major or minor, attributable to the use of direct oral anticoagulants (DOACs) varies based on the introductory trials and, for this reason, the need to stratify patients based on the possible risk associated with this therapy has become widespread. In the RE-LY study [9], which compared dabigatran with warfarin for stroke prevention in atrial fibrillation, the annual rate of major bleeding with dabigatran 150 mg twice daily was approximately 3.11%, while with dabigatran 110 mg twice daily it was approximately 2.71%. Ultimately, the RE-LY study concluded that dabigatran administered at a dose of 110 mg was associated with an incidence of stroke and systemic embolism similar to that found in patients in the warfarin group, compared to a lower incidence of major bleeding. At the 150 mg dosage, it was associated with a lower incidence of stroke and systemic embolism with the same incidence of major bleeding. In the ROCKET AF trial [10], which compared rivaroxaban with warfarin for atrial fibrillation, the annual rate of major bleeding with rivaroxaban was approximately 3.6%, whereas with warfarin it was approximately 3.4%. In the ARISTOTLE study [11], which compared apixaban with warfarin for the prevention of stroke in atrial fibrillation, the annual rate of major bleeding with apixaban was approximately 2.13%, while with warfarin it was approximately 3.09%. In the ENGAGE AF-TIMI 48 study [12], which compared edoxaban with warfarin for the prevention of stroke in atrial fibrillation, the annual rate of major bleeding with edoxaban was approximately 2.75%, whereas with warfarin it was approximately 3.43%. Clinical studies that establish their effectiveness provide valuable information; although these drugs have declared low rates of major bleeding, it has been repeatedly highlighted that the patients tested often belong to a class of individuals at a lower risk of adverse effects compared to the real ones in practice [13]. This does not offer the opportunity to verify the real weight of drug-related adverse events, especially in more complex patients where drug–drug or drug–disease interactions must always be considered [14,15]. With the introduction of some scores such as, for example, the HAS-Bleed in the context of atrial fibrillation, an attempt has been made to classify patients at high or low risk of major bleeding. However, in the case of multipathological and complex patients, these systems often fail to prevent complications [16,17,18,19,20]. A further problem is the misrecognition of conditions with a high risk of bleeding in all patients who should undergo invasive procedures, whose theoretical calculated risk of bleeding may be lower than when corresponding to reality [21]. These are often fragile patients with a precarious state of health who often, as already mentioned, escape the introductory trials of various drugs [22,23]. Therefore, in patients treated with NOACs, a greater stratification of the fragile patient at risk of bleeding necessarily requires knowledge of all the possible clinical conditions at risk of bleeding even if this is not always feasible [24].

## 3. Hemorrhagic Patients and Risk of Ischemic Stroke

**Figure 1:**
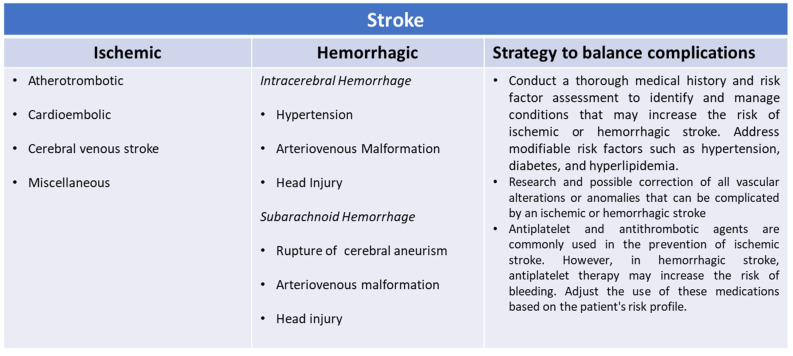
Risk factors for ischemic and hemorrhagic strokes.

As we have already reported, the greater the degree of frailty of the patient, the greater the risk of adverse events in patients at high cardiovascular risk or already undergoing secondary prevention for atherosclerotic or cardioembolic ischemic stroke or with both associated conditions (Figure 1) [21,22,23,24]. The treatment of choice to prevent another ischemic stroke is a long-term oral anticoagulant or antiplatelet strategy. However, many patients cannot take such drugs due to long-term contraindications, comorbidities, cognitive impairment, severe walking difficulties with frequent falls, and patient refusal [25,26]. The risk of death or addiction, as well as the risk of stroke recurrence in this group, is also not well known but must certainly be considered [25,26]. The presence of ongoing bleeding causes a series of problems for patients who, at risk of thromboembolic events, take antiplatelet or anticoagulant therapy [27,28]. However, there is evidence to support the hypothesis that the suspension of these drugs in patients at high cardiovascular and cerebral risk can cause both early and medium-term acute ischemic events related to the hemorrhagic event [29,30,31]. Even if hemorrhagic events and thromboembolic events are antithetical to each other, they show some shared risk factors. Among these, we consider in particular increasing age, frailty, comorbidity, atherosclerosis, genetic predisposition to blood coagulation disorders, and platelet pathology [30,31]. Doctors are therefore faced with a therapeutic dilemma in the management of patients with concomitant hemorrhagic and ischemic pathologies, especially because, at present, there are no systematic reviews and randomized or observational studies that address this problem [32,33]. Most studies conducted in the past did not mention individual modifiable and non-modifiable risk factors, nor the degree of health complexity and fragility of each patient [34,35,36]. It is estimated that the recurrence of a stroke during the secondary prevention period is approximately 20% [37]. Among the first causes, there is certainly the impossibility of carrying out the best treatment or the non-optimal coverage of all the risk factors that can contribute to relapse [38,39]. Patients undergoing secondary prevention after an acute cerebral circulation disorder and with high adherence to therapy show a lower incidence of new events [39,40]. Some studies have hypothesized that low adherence to the optimal therapeutic scheme for the prevention of a further cerebrovascular event is linked to the fear of prescribing drugs with important adverse events, uncertainty about long-term clinical benefits and risks, lack of knowledge and experience in managing such therapies, or competing medical issues such as the cost of medications [41]. Others hypothesize that effective information is lacking in how to limit low drug use in populations with different levels of education or cultural traditions adverse to the use of such important drugs [26,42,43]. However, it is the complexity of the patients that represents the fundamental cause of the unfavorable outcome of the disease [44]. We do not yet have adequate knowledge of this complexity in the field of cerebrovascular diseases. We know that there is already a pre-stroke complexity that affects approximately 25% of the affected population, to which is then added the fragility that follows one or more acute cerebrovascular events [44]. It must be said that stroke is one of the most common acute manifestations observed in the elderly and is related to the majority of systemic cardiac, pulmonary, renal, and degenerative pathologies whose synergistic effect is not yet well known [45]. This is why, despite having cutting-edge therapies for the prevention and treatment of stroke, we have a persistently high mortality and disability rate [45,46].

## 4. Clinical Outcome of Frail Patients

We know that, together with atherosclerosis, atrial fibrillation (AF) is the most important cause of stroke. All three of these conditions become increasingly common in old age and where frailty is greater, characterized by a decline in biological reserves and the deterioration of physiological mechanisms, making the elderly vulnerable to a series of adverse outcome factors [47,48,49,50]. There are little data available on the safety of treatments used in the frail population useful for the primary or secondary prevention of acute cerebrovascular events. In frail patients, a greater number of adverse events related to anticoagulant therapy have been observed, such as bleeding and a greater difficulty in choosing the best therapy for these patients [51]. This leads us to think that the presence of adverse events and the high mortality of these patients is linked precisely to the condition of fragility [52,53]. According to Wilkinson et al. [51], it is precisely bleeding linked to anticoagulant therapy that can be subjected to review in light of the concept of frailty, representing an objective of improving safety and mortality outcomes for this condition. Modifiable hemorrhagic risk factors should be sought and subjected to an optimization process, also in light of the polytherapies often present in these patients [54,55]. Indeed, a dose adjustment of anticoagulant therapy was able to satisfactorily prevent stroke, without a significantly higher incidence of major bleeding compared to placebo [56]. In the following parts, we will discuss what the main factors are that can influence the occurrence of adverse events during chronic treatment for the prevention of stroke or a subsequent event.

### 4.1. Cerebrovascular Anatomical Condition and Hemorrhage and Ischemic Stroke Risk

**Figure 2:**
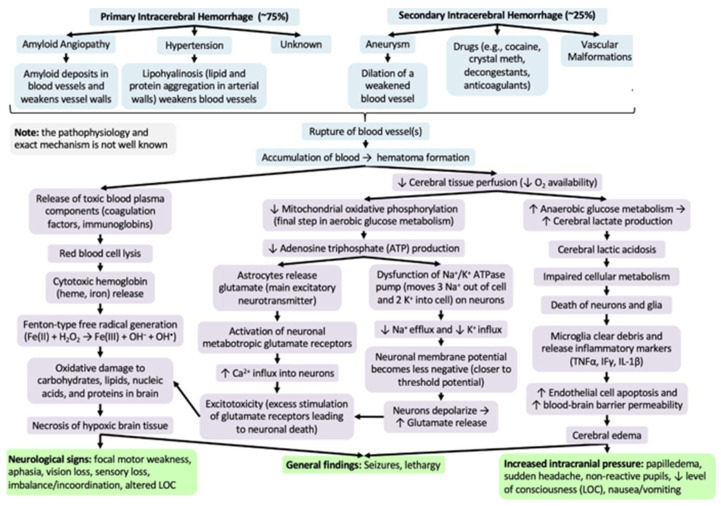
Difference in hemorrhagic stroke.

**Figure 3:**
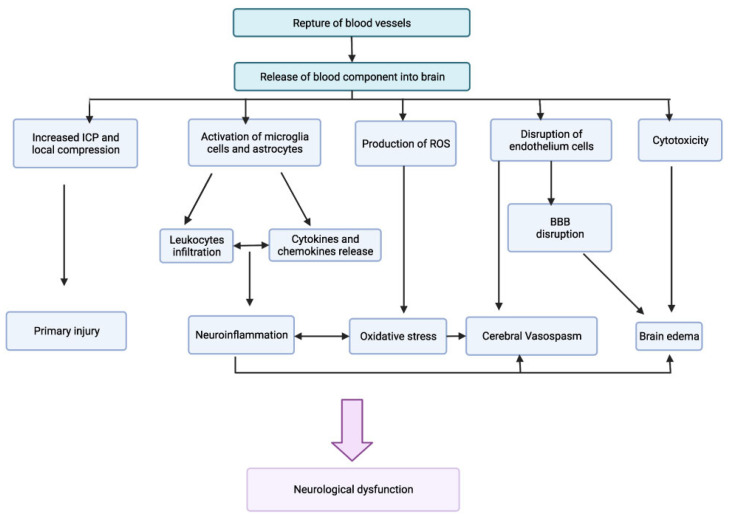
Pathogenesis of rupture of blood–brain barrier *(Adapted)*.

Alterations in the blood–brain barrier (BBB) underlie the manifestations that can result from an intracranial hemorrhage or an ischemic stroke associated with several modifiable cardiovascular risk factors (Figure 2) [57]. In pathological conditions, the loss of the homeostatic microenvironment leads to the impairment of normal neuronal function and the alteration in the permeability of the vascular endothelium [58,59]. During and after ischemic stroke, this phenomenon increases the risk of hemorrhage by creating gaps through the tight junctions of the endothelium [60,61,62,63] (Figure 3). Diabetes and cigarette smoking have been identified as among the risk factors that mostly cause this catastrophic sequence of events, and are associated with the presence of coagulopathy during chronic liver disease and anticoagulant therapy [64,65]. Obviously, patients undergoing anticoagulant therapy are strongly exposed to the risk of intracerebral hemorrhage due to the increased risk of bleeding associated with the use of these drugs [66,67]. Chronic liver disease with associated coagulation disorders is also associated with cerebral hemorrhage but the definitive role of this condition in the hemorrhagic transformation of ischemic stroke has not yet been fully clarified [68]. The latter study in fact associated liver cirrhosis with a high risk of ischemic stroke, venous thromboembolism, and major hemorrhagic complications only in patients suffering from chronic atrial fibrillation [68]. Another study [69] considered that left ventricular dysfunction is also an independent risk factor in the atrial fibrillation of the evolution of cerebral microvascular damage toward lacunar infarction cerebral hemorrhage or a combined form of both conditions. It is very interesting to underline that one study [70] considered how arterial hypertension contributes more to ischemic stroke in patients up to 65 years of age while, in older patients, it contributes more to intracranial hemorrhage.

### 4.2. Size and Severity of the Hemorrhage and Ischemic Stroke Risk

The size and severity of the hemorrhage can influence the risk of complications such as ischemic stroke. Larger hemorrhages involving critical areas of the brain may increase the risk of ischemia [71]. Reperfusion therapies can lead to hemorrhagic transformation in between 3.2 and 43.3% of cases, with a more significant impact on prognosis [72,73]. Etiologically, hemorrhagic transformation is a multifactorial phenomenon, and previous studies, in addition to atrial fibrillation, advanced age, and delay in thrombolytic treatment, have highlighted how the severity of the symptoms and the extension of the ischemic territory are equally important risk factors [74,75,76]. In particular, it should be underlined that the importance of the vessel affected by the occlusion is also crucial in determining the risk of post-procedure hemorrhage [72,77]. Large vessel occlusion worsens stroke severity and is associated with a worse NIHSS score and an involvement of a larger area of brain tissue [78,79]. In the case of a cardioembolic stroke, the numerousness of the clots was predictive of the overlap of a hemorrhagic complication considering the possibility that, in this case, there could be a greater extension and a multiplicity of the infarcted brain areas [72,80]. Iancu et al. [81] looked for risk factors and hemorrhagic transformation in patients with an initial diagnosis of ischemic stroke. Among these, the presence of male sex, high glycemic values of arterial hypertension at baseline, and high values of NIHSS and ASPECTS scores were all associated with a significantly elevated risk of hemorrhagic transformation [81]. In this study as in others, the assessment of stroke severity and early detection of tomographic characteristics of the infarcted ischemic lesion were the main predictive factors of hemorrhagic transformation [82,83]. In particular, it has been seen that a score above 20 is considered to be at high risk of hemorrhagic transformation regardless of whether the cerebral arterial occlusion has undergone revascularization or not. Another study, however, does not recommend mechanical thrombectomy in the presence of an NIHSS score ≥20 due to the high possibility of hemorrhagic events [84]. Another interesting study found a correlation between an increased NIHSS score and the concomitant presence of atrial fibrillation and tumors [85]. These data regarding the concomitant presence of atrial fibrillation have not been confirmed in other studies [81], while, with regard to the diagnosis of a concomitant neoplasm, it is difficult to find case series that evaluate patients also for the purposes of researching a neoplasm occult. To conclude, a review demonstrated that the occurrence of hemorrhage in the context of an ischemic area depends on many factors, including the location and size of the infarct and poor collateral vascularization, confirming what has been said previously [86]. Therefore, by reflecting on these factors, in the future, one will be able to better stratify the risks before treating a patient with a stroke to avoid worsening the prognosis.

### 4.3. Individual Risk Factors in Patients with Hemorrhagic Transformation 

The presence of independent risk factors such as arterial hypertension and diabetes mellitus strongly contributes to the worsening of the prognosis in these patients. The main studies [87,88] that have correlated diabetes mellitus with a greater severity of the stroke and therefore a greater risk of hemorrhagic transformation have highlighted that glycemic decompensation has a negative effect on the BBB and is associated with negative prognostic outcomes. The impact of glycemic imbalance on the metabolism of neurons affected by the stroke is biphasic: first, it is a protective factor; then, it represents the optimal condition for the formation of oxygen free radicals that alter the balance of the barrier, leading to a reduced reperfusion of the microvascular circulation, edema of endothelial and cerebral cells, and therefore hemorrhagic transformation, complicating the clinical picture [89,90,91]. The same destabilizing effect on the BBB has been observed for blood pressure as the latter is also capable of causing its permeability [92,93,94,95,96]. In patients with a long history of arterial hypertension, the small vessels of the cerebral circulation exhibit stable endothelial damage that further aggravates the inflammatory damage caused by circulating oxygen free radicals [92,93,94,95,96]. It must also be said that the ischemic stroke itself causes an increase in blood pressure values that essentially derive from the type of stroke, the state of recanalization, and the reorganization of the flows through the collateral vessels [97]. In the case of a hemorrhagic stroke, the reduction in blood pressure values can help to limit the extent of the blood share but does not influence functional outcomes [98]. It is therefore possible that blood pressure is not sufficiently controlled alone, but may influence the outcome of treatment in synergy with other measures [95]. AF is associated with an increased risk of intracranial hemorrhage, although this is largely related to concomitant anticoagulant therapy. In a limited number of cases, it has been shown that patients with atrial fibrillation can develop extracranial hemorrhage (ECH) [99]. However, it is unclear whether patients with AF may have a better or worse prognosis than those without arrhythmia [100]. Patients with AF certainly benefit from thrombolytic treatment within the established timeframes, with some associated bleeding risk. Furthermore, it is reiterated that the treatment of all predisposing conditions is truly associated with the favorable outcome of treatments in ischemic stroke. One study [100] also considers that, generally, AF patients suffering from ischemic stroke are significantly older and have a higher NIHSS score than the non-AF group. Therefore, it is necessary to consider that patients with AF are at greater risk of adverse events and poor prognosis, regardless of concomitant anticoagulant therapy [100]. Atherosclerosis is to be considered as a condition that can alternatively cause ischemic or hemorrhage, so its progression is strongly associated with possible dual manifestations of thrombosis and hemorrhage [101]. Intracranial hemorrhage is the only pathological form that unites some rather rare conditions, including cerebral amyloid angiopathy, reversible cerebral vasoconstriction syndrome, and reversible posterior leukoencephalopathy syndrome [102]. The coexistence of intracranial hemorrhage is often described as a complication of acute ischemic stroke without this association being fully understood [103,104,105]. It is known that, in these cases, half of the patients had severe stenosis or occlusion of the large arteries [103,104,105,106,107]. All these conditions are secondary to degeneration at the level of the stenosis, which causes rupture of the fragile dilated collateral vessels, accompanied by emboli that enter the marginally perfused vessels, leading to necrosis and rupture [108,109].

### 4.4. Pathological Condition and Risk of Ischemic or Hemorrhagic Stroke

Several studies gathered in a meta-analysis [110] have reported that cirrhosis, especially cirrhosis in the decompensation phase, can increase the risk of stroke. In general, an increase in total strokes was reported, which were largely hemorrhagic strokes. It is known that liver cirrhosis, especially decompensated cirrhosis aggravated by liver failure, can cause defects in the blood coagulation structure, exposing patients to the risk of bleeding, largely linked to the lengthening of clotting times and the sequestration of platelets in the hepatosplenic circulation, but also thrombotic complications due to the defect in the production of proteins with a natural anticoagulant function (protein C, protein S, and antithrombin III) [111]. A meta-analysis, subsequent to that cited previously, aimed to establish the potential association between liver cirrhosis and the various forms of acute cerebrovascular accidents such as hemorrhagic stroke, ischemic stroke, intracranial hemorrhage, and subarachnoid hemorrhage. The study highlighted how, in cirrhotic patients, there was a significant increase in intracranial and subarachnoid hemorrhage compared to both forms of stroke [111]. In this regard, the authors concluded that it was necessary on a case-by-case basis to follow the blood coagulation status over time and subject the patient to suitable prophylaxis [111].

Patients with hematological diseases often experience cerebrovascular complications including ischemic stroke, intracerebral and subarachnoid hemorrhage, and microbleeds [112]. Hemorrhagic stroke is a very rare occurrence in chronic myeloproliferative diseases but when it occurs it can be fatal [113,114]. Particularly in forms such as polycythemia vera and essential thrombocytosis, ischemic stroke is frequent. In these cases, cytoreductive therapy and antiaggregants may be taken into consideration in high-risk patients [115,116]. The leukemic forms, especially the acute ones, can cause ischemic or hemorrhagic stroke, sometimes presenting themselves as the onset of the hematological pathology [117]. In these cases, stroke is linked to the state of hypercoagulability, disseminated intravascular coagulopathy (DIC), leukocyte stasis linked to the proliferation of white blood cells, and forms of endocarditis with platelet vegetations associated with paradoxical embolism [117]. The AF and other traditional vascular risk factors, infections, and anti-tumor therapies (radiotherapy, chemotherapy, immunotherapy, and transplantation) can be additional risk factors causing stroke. Intracranial bleeding is a serious, potentially fatal cerebrovascular complication in patients with acute leukemia [112]. Bleeding can occur in one or more locations, mainly linked to thrombocytopenia. The presence of multiple hemorrhagic foci of hemorrhage, thrombocytopenia, leukocytosis, and the early presence of a comatose state has been considered as the most unfavorable prognostic factors for survival [118]. The leukemic form with the highest hemorrhagic risk is acute promyelocytic leukemia, which is often associated with disseminated vascular coagulation and fibrinolysis (with alternating sectoral forms of a thrombotic or hemorrhagic type at the same time) [119]. The same mechanisms listed above are common to all other forms of stroke that also occur in lymphomas. In these latter forms, the presence of vasculitis, thrombotic microangiopathy, and blood stasis induced by compression and ab extrinsic of the lymphatic packets can be further factors causing hypercoagulability and therefore stroke [120]. An increase in ischemic or hemorrhagic risk is also reported in patients with multiple myeloma linked in particular to hypercoagulability, hyperviscosity, thrombotic microangiopathy, and embolism [121,122,123]. Furthermore, many drugs used for these pathological forms can have thrombogenic side effects [124]. Thrombotic and thrombocytopenic purpura can cause ischemic or hemorrhagic strokes even at a young age, usually with multifocal onset with involvement of the large arteries [125]. Approximately half of the patients presented neurological forms associated with hemolysis and thrombocytopenia [125].

### 4.5. Therapies and Treatments Influences in Patients with Hemorrhagic Transformation

**Figure 4:**
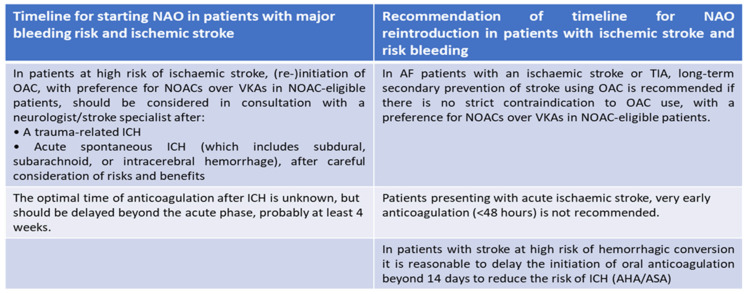
The optimal strategy to reintroduce NAO in therapy according to standard guidelines 2020 ESC Guidelines for the diagnosis and management of atrial fibrillation (European Heart Journal 2020-doi/10.1093/eurheartj/ehaa612).

The bleeding risk is known as the main problem of all anticoagulant therapies, despite their proven effectiveness [99]. The introduction of direct oral anticoagulants (DOACs) into clinical practice means that major hemorrhages, especially intracranial, associated with therapy are certainly reduced; however, the risk of bleeding, even with these drugs, is not eliminated. Hence, there is a need to develop new drugs that are ideally capable of overcoming the current limitations of therapies in use (Figure 4) [99]. In particular, it would seem that the risk increases in conjunction with acute renal failure as all direct and indirect anticoagulant drugs have a predominantly renal excretion mechanism and are therefore subject to hyperaccumulation with the possibility of an increase in major and minor hemorrhages [126]. It has been shown that the frequency of cerebral hemorrhage is significantly increased in patients with chronic renal failure, with a significant increase in deaths during dialysis [127]. Although the data available to us showed that DOACs reduced the risk of major bleeding compared to VKAs in patients with chronic renal failure, especially in patients with atrial fibrillation, the risk of major hemorrhagic events is concrete [128,129]. The heterogeneity of the various studies makes it difficult to understand whether in the presence of renal failure the prevention of stroke has, in terms of safety results, entailed the same risk of bleeding compared to not carrying out anticoagulant therapy. An increase in intracranial hemorrhage with the use of anticoagulants was a notable finding. For patients with atrial fibrillation and end-stage renal disease, there is a clinical equilibrium in which the risk of bleeding with any anticoagulant may outweigh any potential benefit [130,131].

Patients at risk of ischemic stroke related to cardioembolic situations may be at risk of stroke despite the use of correct anticoagulant therapy [132]. In most cases, this phenomenon, which involves both large and small arteries, is linked to the atherosclerotic process and to the various nuances that characterize cryptogenic forms [133]. However, if anticoagulation is correctly performed, the optimization of secondary prevention therapy may require integration with antiplatelet therapy to make the treatment more effective in preventing major adverse cardiovascular events [134,135]. However, on the contrary, other data suggest that the addition of antiplatelets to anticoagulants provides no benefit in patients with atrial fibrillation and stroke and increases the long-term risks associated with combined anticoagulant and antiplatelet treatment in patients with atrial fibrillation [136].

## 5. Ischemic and Hemorrhagic Stroke in COVID-19 Patients

During the peak of the COVID-19 pandemic, an increase in acute neurological disorders of cerebral circulation was observed, especially in patients hospitalized in intensive care units [136,137,138]. These events were mainly of a thrombotic nature but a non-negligible number of cerebral hemorrhagic manifestations was also highlighted [139,140]. The establishment of a state of hypercoagulability during COVID-19 infection seems to play a decisive role in these manifestations so, in these patients, it was carried out early with pharmacological prophylaxis of venous thromboembolism (VTE) and directed against the excessive tendency to induce platelet aggregation [141,142,143,144,145]. Intracranial hemorrhage (ICH) is less frequent than ischemic strokes in COVID-19 patients. However, ICH is associated with a high mortality rate [146]. At the beginning of the pandemic, Sharifi-Razavi et al. [147] wondered whether ICH was a direct effect of COVID-19 infection or the consequence of prophylactic anticoagulant treatment, and other studies have shown that these events are associated with a loss of the integrity of the BBB and with an increase in inflammatory cytokines (IL-1, IL-6, TNF-α) [148,149]. Finally, as previously mentioned, a state of hypercoagulation can also produce thrombotic microangiopathy of small vessels, loss of endothelial integrity, and subsequent rupture [150].

## 6. Conclusions

When treating patients with neurological manifestations related to an ischemic or hemorrhagic stroke, any underlying conditions or possible consequences related to the modulation of the hemocoagulatory and fibrinolytic system must always be taken into consideration. Patients with AF and a higher NIHSS score, diabetics, and elderly patients should be considered in the highest risk category for the probable evolution of ischemic stroke to hemorrhagic stroke. Hemorrhagic transformation in the different contexts in which it can occur is one of the complications that most influence the good outcome in terms of functional prognosis and in terms of survival. It is mainly due to an alteration in the BBB with a consequent alteration in endothelial permeability and inflammatory damage. The same etiopathogenesis is also common to those cases related to COVID-19 infection, where the inflammatory cascade linked to the circulating virus plays an essential role in modifying the endothelial balance. Future studies should be aimed at improving knowledge on the management of anticoagulant therapy in fragile patients, perhaps with the introduction of more selective anticoagulant drugs with less hemorrhagic impact than those still available. Another direction of study is to acquire further knowledge on the correct timing of the suspension and reactivation of anticoagulant therapy in high-risk patients. In this field, there are still gray areas, especially when it comes to recovery after a major hemorrhagic event and when it comes to any factors that can favor the appearance of complications.
